# The Current Directions of Searching for Antiparasitic Drugs

**DOI:** 10.3390/molecules27051534

**Published:** 2022-02-24

**Authors:** Katarzyna Dziduch, Dominika Greniuk, Monika Wujec

**Affiliations:** Department of Organic Chemistry, Faculty of Pharmacy, Medical University of Lublin, 4A Chodzki Street, 20-093 Lublin, Poland; katarzynadziduch@o2.pl (K.D.); d.greniuk95@gmail.com (D.G.)

**Keywords:** antiparasitic activity, drugs, new chemical compounds

## Abstract

Parasitic diseases are still a huge problem for mankind. They are becoming the main cause of chronic diseases in the world. Migration of the population, pollution of the natural environment, and climate changes cause the rapid spread of diseases. Additionally, a growing resistance of parasites to drugs is observed. Many research groups are looking for effective antiparasitic drugs with low side effects. In this work, we present the current trends in the search for antiparasitic drugs. We report known drugs used in other disease entities with proven antiparasitic activity and research on new chemical structures that may be potential drugs in parasitic diseases. The described investigations of antiparasitic compounds can be helpful for further drug development.

## 1. Introduction

One might suppose that the problem of parasitic diseases does not concern us anymore, but nothing could be more wrong. Despite the great knowledge about parasites and the fight against them, as well as the improvement of hygiene conditions, we can observe the return of problems related to parasitosis. Along with the popularization of tourism, population migration and civilization changes, parasitic diseases appear in places that have not been observed so far.

The most common parasitic diseases are those caused by soil-borne worms (helminths). The World Health Organization estimates that approximately 24% of people worldwide may be infected with soil-borne parasites [1].

Climate change also causes more parasitic infections. The life cycles of parasites and the transmission of pathogens to humans, domestic and wild animals are inextricably linked with climatic conditions. In the northern regions of Europe, climate change changes the populations of parasites, their hosts and vector species. Currently, parasites that previously could not survive in these areas started to spread in this part of the continent, and thus, new diseases appear [2].

The COVID-19 pandemic has led to an increase in the immunosuppressed population worldwide due to both disease and corticosteroid use. This, in turn, increased the risk of opportunistic parasitic infections. On the other hand, the lack of communication during a pandemic and irregular drug deliveries to African countries may significantly increase the number of parasitic infections.

Parasites also put at risk animal productivity and food production. Over 500 million large ruminants are infected with parasitic worms, which results in economic losses of over $3 billion p.a. worldwide [3]. It is also worth remembering that together with parasites, they carry various types of microorganisms (viruses, bacteria, fungi) that can cause serious infections.

Despite so many dangers, pharmaceutical companies are generally not interested in researching new antiparasitic drugs. Until now, drugs that were invented in the last century have been used alone or in combination therapy. Suramin, a drug invented in 1920, is still used in trypanosomiasis.

Antiparasitic drugs have many side effects and are relatively toxic. Moreover, they are often not effective. This is because the parasites become resistant to the used drugs. This is a huge problem. Therefore, new structures, “not recognized” by parasites, are constantly searched for.

An alternative to synthetic compounds may be natural compounds obtained from plants. There are many well-known plant oils with antiparasitic activity. The most important plants are *Allium sativa*, *Melissa officinalis*, *Origanum vulgare*, *Thymus vulgaris*, *Cinnamomum zeylanicum*, *Melaleuca alterinfolia*, *Citrus limon*, *Xylopia aethiopica*, *Cochlospermum planchoni*, *Virula surinamensis, Nigella sativa* [4,5,6,7,8,9,10]. Unfortunately, most of them show many toxic effects, including the cytotoxic effect on mammalian cells, which significantly limits the possibility of their use. However, some plants like *Morus alba* contain compounds with biological activity: antimicrobial, antioxidant, antidiabetic, anxiolytic, and anthelmintic [11,12]. Mirazid is a new natural antischistosomal drug formed from myrrh extract. It seemed to be safe, unlike praziquantel, an antiparasitic drug [13].

There are relatively few reports in the literature on new scientific research on antiparasitic agents. Attempts are made to find effective antiparasitic drugs among the drugs already available for other diseases, and new compounds with potential antiparasitic activity are synthesized.

This manuscript presents the current directions for searching for compounds with antiparasitic activity.

## 2. Analogs of Popular Drugs

### 2.1. Linezolid Analogs

Linezolid is an antibiotic used in the treatment of nosocomial infections caused by multiresistant strains of gram-positive bacteria. Moreover, it has proven activity against *Mycobacterium tuberculosis*. The structural basis of the compound is 1,3-oxazolidin-2-one [14]. Alcántar-Zavala et al. synthesized six new linezolid analogs (Table 1), which in vitro are a promising and potentially effective group of compounds that can be used in the treatment of *Hymenolepis nana* tapeworm infections. The new compounds were received by the chemical modification of L-alanine. First of all, the synthesis of diastereomeric N,N-dibenzylamine oxazolidinones and their coupling with 4-(4-bromophenyl)morpholine were important. The aim of this activity was to obtain analogs of N,N-dibenzylaminolinezolid (**4** and **5**). Then, in the hydrogenolysis reaction (compounds **4** and **5**), amino acid-free analogs of linezolid (**6** and **7**) were obtained, which were subjected to an acylation reaction to obtain diastereomeric derivatives of linezolid (**8** and **9**).

The researchers found that linezolid alone does not cause damage to anatomical parasites, but the reverse, causing paralysis of the parasites and consequently there is a risk of the survival of the parasite after 24 h of treatment. The great potential of the newly synthesized analogs of linezolid is due to their enhanced activity. Compared to praziquantel, linezolid analogs cause anatomical damage to the parasite and are not cytotoxic in vitro.

Among the newly obtained compounds, the most potent compounds are: (R)-5-((S)-1-aminoethyl)-3-(3-fluoro-4-morpholinophenyl)oxazolidin-2-one, (S)-5-((S)-1-aminoethyl)-3-(3-fluoro-4-morpholinophenyl)oxazolidin-2-one (**6** and **7**), ((R)-((S)-3-(3-fluoro-4-morpholinophenyl)-2-oxooxazolidin-5-yl)ethyl)acetamide, and ((S)-((S)-3-(3-fluoro-4-morpholinophenyl)-2-oxooxazolidin-5-yl)ethyl)acetamide (**8** and **9**). They are characterized by a shorter time of paralysis and death of the parasite after administration at the dose of 20 mg/mL (6–10 and 18–20 min). In the case of praziquantel, the time is longer and amounts to 20 and 30 min at the dose of 20 mg/mL. Analogs **4** and **5** induced paralyzes and death at 20 and 60 min, respectively. Although compounds **4** and **5** show the death of the parasite twice as long as praziquantel (30 min), the interesting fact is that they cause damage to the sacrum, neck and parasite proglottids.

According to the literature, oxazolidine compounds are one of the first reports of linezolid derivatives showing antiparasitic activity [14].

### 2.2. Amiodarone Analogs

The only available treatment option for Chagas disease caused by *Trypanosoma cruzi* is chemotherapy with the use of nitroaromatic compounds, nifurtimox and benznidazole. Unfortunately, it was found that both compounds did not bring the desired effects in people with chronic diseases [15]. Clinical trials with benznidazole have shown that it does not reduce the cardiomyopathy that can occur in patients infected with the parasite. Hence, in recent years, researchers are determined to search for new compounds effective against *Trypanosoma cruzi* [16]. Amiodarone is an antiarrhythmic drug with a benzofuran ring in its structure. It has been known since the 1970s that compounds with benzofuran in their structure, as well as their analogs, are effective in the treatment of *Trypanosoma cruzi* infections [17].

It has been shown that amiodarone, in addition to cardiac activity, also has antiparasitic properties. The effect of amiodarone on *Trypanosoma cruzi* has been described for the first time. Strong synergism of this compound with posaconazole has been found. Its effectiveness against *Leishmania mexicana* has also been confirmed. The synergism of action with miltefosine has been demonstrated [18].

The iodine atom in the structure of amiodarone causes many side effects of this substance. The amiodarone analog, which has been deprived of iodine, is dronedarone. As a result, it has fewer side effects and shows safer operation. Similarly, to amiodarone, it showed effectiveness against both *Trypanosoma cruzi* and *Leishmania mexicana*, with greater safety and fewer side effects. The main mechanisms of action of amiodarone and dronedarone are the inhibition of ergosterol synthesis, a disorder of calcium homeostasis in parasite cells [19].

**Figure 1:**
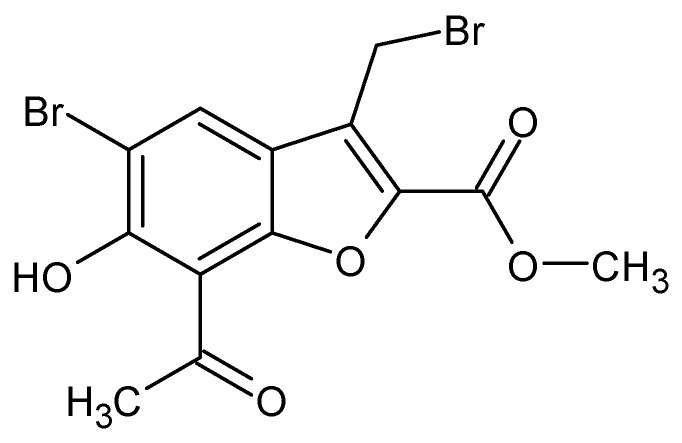
The structure of amioder.

In the article: *Anti-Trypanosoma cruzi action of a new benzofuran derivative based on amiodarone structure*, a new action of the benzofuran derivative, amioder, was described (Figure 1). In order to determine the effectiveness of the drug, studies were performed to assess the effect of the substance on cells infected with *Trypanosoma cruzi*. Amastigotes were grown in Vero cells in 24-well plates. The next study was to determine the intracellular calcium concentration of *Trypanosoma cruzi* epimastigote using the radiometric Fura 2 index. The potential of the mitochondrial membrane was also determined using a fluorescent dye, rhodamine 123 [20].

Amioder shows antiparasitic activity inhibiting the growth of the parasite. The calculated IC_50_ value was 8.5 µM. Assessing the effect of the analogs of amiodarone on *Trypanosoma cruzi* infected cells using the cell lines (Vero cells), it was found that amioder reduce the overall percentage of infected cells (IC_50_ = 1 µM) at low cytotoxicity to normal cells (IC_50_ = 39.5 µM).

In summary, Pinto-Martinez et al. proved that amioder inhibits the growth of *Trypanosoma cruzi* epimastigotes and amastigotes in host cells. This makes it a good potential antiparasitic drug [20].

### 2.3. Ergotamine Analogs

Chan et al. found that the ergotamine so far used in the treatment of migraine interacts with a protein that controls parasite motility (*Schistosoma*). Compared to praziquantel, ergotamine, in addition to eliminating parasites, also limits the number of organ damage caused by infection, including protection against liver and spleen enlargement. However, due to numerous side effects occurring after repeated administration of ergotamine, there is little chance to use this drug in people with schistosomiasis. Knowledge of the novel actions of this substance could allow scientists to identify and test new compounds with similar properties and a more favorable safety profile [21].

### 2.4. Auranofin

Auranofin is a drug used in patients with rheumatoid arthritis. It has few side effects. Patients treated for 6 months with auranofin did not report serious adverse effects. Most often, they had gastrointestinal complaints—diarrhea. Previous studies have shown that this drug is an effective antiparasitic agent against a number of organisms, including *Plasmodium falciparum* [22], *Echinococcus granulosus* [23], *Schistosoma mansoni* and *S*. *japonicum* [24], *Taenia crassiceps* [25], *Leishmania* spp. [26], *Trypanosoma brucei* [27], *Giardia lamblia* [28] and *Toxoplasma gondii* [29]. The target of auranofin in *Brugia* is probably thioredoxin reductase, but its mechanism of action in parasitic diseases has not yet been well studied. Research shows that auranofin was effective in vitro in killing both female and male *Onchocerca* and *B. malayi* worms. Currently, the substance has been recognized as a potential antiparasitic agent, being effective in the treatment of lymphatic filariasis and onchocerciasis [30].

### 2.5. New Activity of Promethazine

Promethazine is a phenothiazine derivative, an H1 receptor antagonist, belonging to the group of antihistamines. Mainly promethazine has sedative, anticholinergic, antiemetic and local anesthetic properties. Newly conducted ex vivo studies on promethazine indicated its antiparasitic activity against adult forms of *Schistosoma mansoni*. The experiment also proved that after the use of promethazine, the motor activity of the parasite in the venous plexuses of the intestines of an infected patient is reduced, and the movement of the female and male suckers is impaired. In studies in murine models, the substance has fatal effects on juveniles and adults of *S. mansoni* and a reduced risk of enlargement of the liver and spleen, which may be due to infection. Moreover, the action of promethazine is superior to the equally interesting drugs, such as artesunate, artemether, mefloquine and miltefosine [31].

## 3. Currently Used Drugs with Newly Discovered Antiparasitic Properties

### 3.1. Ivermectin

Ivermectin is a macrocyclic lactone that exhibits a broad spectrum of antiparasitic activity. It is effective in onchocerciasis; the disease caused by the *Loa loa* nematode and in lymphatic filariasis [32]. It is used to treat and control soil-transmitted helminths, strongyloidiasis [33]. In Australia, the substance is also approved for the treatment of scabies. It is also indicated in the treatment of crusted scabies. Additionally, ivermectin is also indicated as a second-line therapy for typical scabies where topical permethrin has failed. It is also used to treat typical scabies when topical permethrin is not effective [34,35].

Ivermectin affects microfilaricides, which means that it shows no mortality in adult forms, but only in their larval forms. Therefore, treating patients with ivermectin should last for several years until the adult worms die naturally. In order to increase the effectiveness of the treatment, a combination of preparations is used. An example of an effective polytherapy in the treatment of *Dirofilaria immitis* is the combination of ivermectin with doxycycline. Thanks to this therapy, it brings about the death of microfilariae with the help of ivermectin and microfilariae using an antibiotic from the tetracycline group. In veterinary and medicine for the treatment of parasitic diseases, one of the most commonly used drugs is ivermectin.

In addition, it has been proven that blood containing ivermectin is deadly against mosquitoes. For this reason, the use of ivermectin may be a new strategy for reducing the transmission of malaria [36].

### 3.2. Diethylcarbamazine

Diethylcarbamazine is used in nematode infections, often in combination with albendazole. It has a lethal effect against the larvae of parasites. The substance is used to control lymphatic filariasis in countries where there are no co-infections with *Onchocerca volvulus* (except Africa). A significant problem in the treatment of filarial disease is the risk of developing drug resistance. There is also no macrofilaricidal drug. Diethylcarbamazine is a microfilicide with an unknown mechanism of action. However, there are studies confirming its macrofilaricidal activity by influencing the metabolic pathways of arachidonic acid and nitric oxide [37] or changes in the host immune system [38]. The substance is especially effective against *Loa loa* [39].

### 3.3. Moxidectin and Selamectin

Moxidectin and selamectin are macrocyclic lactones used in canine heartworms. They are lethal against *Dirofilaria immitis L3* and *L4*. Their activity is to inhibit the life cycle of the parasite.

Moxidectin is a substance approved in 2018 for the treatment of onchocerciasis for people over 11 years of age in the United States. Previously, it was used in veterinary medicine. Compared to ivermectin, moxidectin shows a longer half-life in plasma, greater efficacy, and a safer profile of action. Additionally, it can be used in parasitic infections in ivermectin-resistant animals [40].

### 3.4. Flubendazole

Flubendazole is a benzimidazole derivative of methylcarbamate, which was designed and manufactured as a substance for the treatment of canine and feline gastrointestinal nematodes. In the tests, the substance showed a lethal effect against filaria. In Europe, flubendazole has been approved for human use. Due to its limited solubility in water, flubendazole administered orally in the form of a suspension or tablets showed low efficacy. The marked improvement of flubendazole systemic availability was observed after the administration of the cyclodextrin-based formulation. It was characterized by increased bioavailability in tissue and blood in the treatment of onchocerciasis and lymphatic filariasis. Currently, this substance is considered to be one of the most effective macrofilaricidal drugs among benzimidazoles and has great potential in the treatment of filariasis [41].

**Figure 2:**
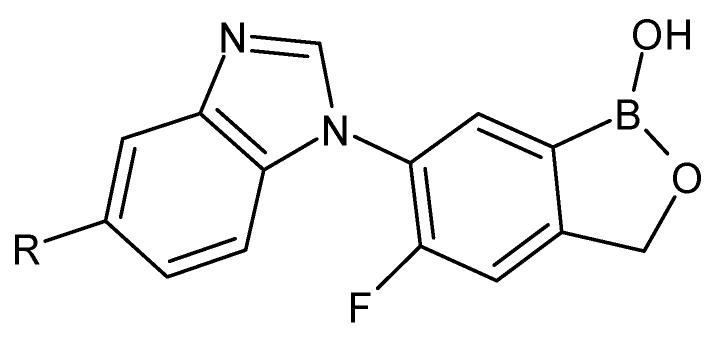
The structures of benzoxaborole analogs of flubendazole.

As a result of limited solubility and embryotoxicity after the use of flubendazole, analogs of benzoxaborole-benzimidazole were synthesized. These compounds have been reported by the pharmaceutical company Anacor (now Pfizer) (Figure 2).

**Figure 3:**
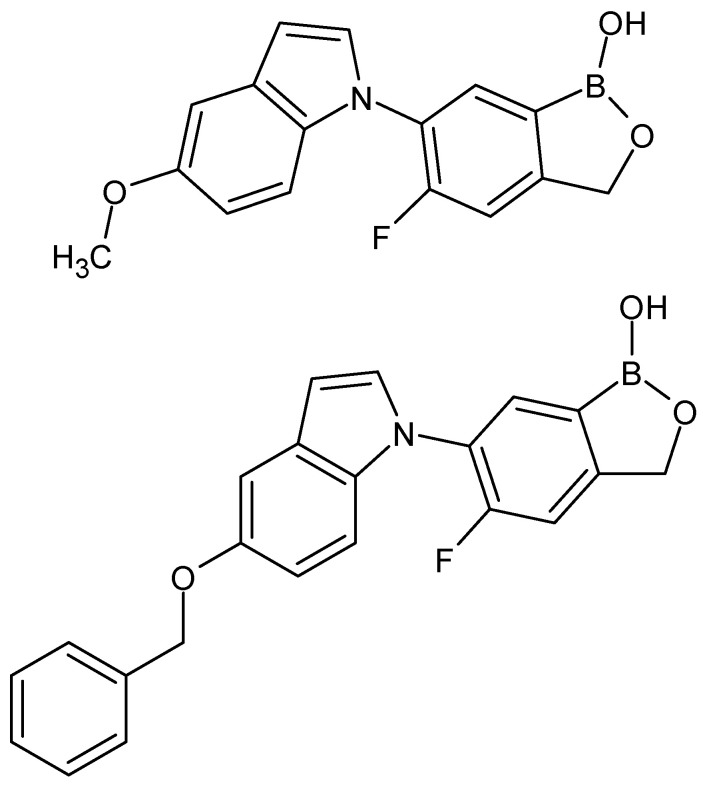
The structures of benzoxaborole analogs with indole moiety.

The same authors described the synthesis of compounds that have indole instead of the benzimidazole ring. These two compounds are the most potent substances causing the death of all tested parasites within 24 h (Figure 3) [42].

**Figure 4:**
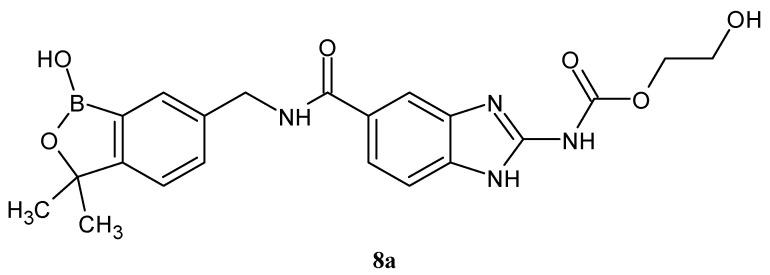
The structures of benzimidazole derivatives.

Compound **8a**, belonging to the benzimidazole group (Figure 4) with an amide bond, showed positive pharmacokinetic properties. After subcutaneous administration as a suspension at a dose of 100 mg/kg/day for 14 days, it showed effective antiparasitic activity against *Brugia malayi*, *Brugia pahangi* and *Litomosoides sigmodontis* [42].

### 3.5. Oxfendazole

Oxfendazole is a benzimidazole derivative with a broad spectrum of antiparasitic activity. It is a drug approved for use in veterinary medicine. The announcement is a new use of this drug in the treatment of onchocerciasis. In preclinical studies, oxfendazole has shown significant antiparasitic activity in the treatment of neurocysticercosis and intestinal parasites. Oxfendazole exhibits lipophilic properties. It is characterized by low solubility in water. Its bioavailability can be increased through the consumption of high-fat foods. It has been shown in studies that an increase in the dose of oxfendazole leads to a decrease in its bioavailability. The drug was well tolerated by the participants in the study, regardless of their gender. No significant side effects were recorded after the administration of the drug 43.

Based on the research, it was found that oxfendazole shows macrofilaricidal effectiveness both after oral and subcutaneous administration. Compared to flubendazole, it has better pharmacokinetic properties. Oxfendazole shows a much better bioavailability than flubendazole after oral administration [44].

### 3.6. Emodepside

Emodepside is a cyclooctadepsipeptide that is used to treat parasitic diseases in animals. Currently, clinical trials are underway to use this substance in the treatment of helminthiasis in humans. It exhibits a different mechanism of action than available drugs, therefore it is an interesting direction of research in the treatment of onchocerciasis. In clinical studies, when the substance was administered to healthy volunteers, emodepside was found to be a safe and well tolerated novel modulator of BK/SLO-1Ca^2+^activated potassium channels in humans, nematodes and insects. Preliminary studies show that emodepside has a good safety profile at a dose of 40 mg and was well tolerated at a dose of 20 mg by volunteers who participated in the study [45].

### 3.7. Triclabendazole

Triclabendazole is the only highly effective drug used in the treatment of human fascioliasis. Increasing resistance to this substance has been noticed in farm animals that have been massively treated with triclabendazole, which may be a potential hazard for humans in the future. So far, no case of resistance in humans has been reported.

New methods of treating fascioliasis are designed to limit the increase in drug resistance of the parasite. For this purpose, combinations of triclabendazole with other drugs, i.e., artemether and attenuate, are used. Due to this combination, a synergistic effect in the control of resistant parasites has been obtained. It should be emphasized that the effectiveness of artemether has not been confirmed in human clinical trials. It was estimated that after administration of artemether to patients infected with the liver fluke, the percentage cure was 6% to 35% [46].

## 4. New Compounds with Potential Antiparasitic Activity

### 4.1. Analogs of Bisnaphthalimidopropyl (BNIP)

The drug of the first choice in the treatment of leishmaniasis is meglumine antimonate. The following drugs are used as alternative treatments: amphotericin B, paromomycin and pentamidine. These substances cause many side effects, increase resistance and the risk of toxic effects [47].

Chagas disease is caused by *Trypanosoma cruzi*. The therapy is based on the use of two drugs, nifurtimox and benznidazole. As a result of many serious side effects occurring after the implementation of treatment based on the administration of the above substances, there is a need to develop new, safer and effective drugs to combat Chagas disease and also leishmaniasis [48].

**Figure 5:**
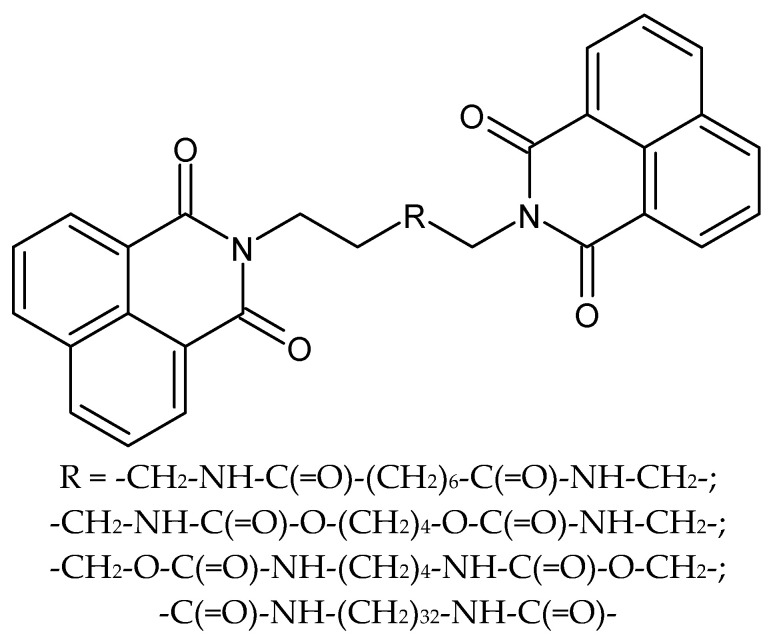
The structures of new analogs of bisnaphthalimidopropyl.

Recent studies have proven the high antiparasitic activity of bisnaphthalimidopropyl derivatives composed of two naphthalimide groups which are linked with a polyamine chain (Figure 5). They work by selectively affecting sirtuin proteins and also by interacting with DNA. Despite the confirmed results of research on anthelmintic activity, the compounds are too toxic to human macrophages and are characterized by low solubility. For this reason, Elif Keskin et al. have received a series of new analogs that differ in the presence of alkyl linking chains. As a result, compounds with better solubility and antiparasitic activity were obtained. Currently, BNIP derivatives are being tested for antiprotozoal activity against *Leishmania infantum* [49].

### 4.2. Analogs of Trifluoromethylated Hybrids of Pyrazole

Compounds based on the pyrazole ring may represent a promising form for the discovery of new antiparasitic drugs. In 2015, researchers discovered the effectiveness of heterocyclic hybrids containing a pyrazole moiety in combating *Leishmania aethiopica* [50]. In turn, in 2019, the effectiveness of pyrazole derivatives against *Trypanosoma cruzi* was proven [51].

Camargo et al. synthesized eighteen new trifluormethylated pyrazole hybrids (Table 2) and tested for efficacy against *Trypanosoma cruzi* and also *Leishmania amazonensis*. The toxicity of all compounds was assessed using the cell lines: LLCMK_2_ epithelial cells and J774A1 macrophages. IC_50_ values for compounds **2a**–**f** were in the range of 18.9–61.7 µM relative to *Leishmania amazonensis*. The most active compound had a bromo substituent on the aromatic ring. Compound **2a**, for which the IC_50_ value was 26.7 µM, with the electron-accepting nitro substituent, and compound **2f** with the electron-donating methoxy substituent in the *para* position, for which the IC_50_ value = 22.4 µM, were characterized by successively high activity. The selective index for macrophages was between eight and nine. In the case of **3a**–**f** compounds containing the following substituents: nitro, methoxy and bromo in the *para* position, the IC_50_ values were: 18.3, 14.2 and 13.9 µM, respectively, which proves their high antiparasitic effectiveness. Increasing the activity, even 1.5 times in relation to the compounds from the second series, was achieved by carrying out the methylation of the sulfur atom. The compound with the highest activity turned out to be derivative **3d**, for which the selectivity index was 10.55 in relation to macrophages. From the group of compounds **4a**–**f**, the substances **4a**, **4d** and **4f** showed the highest antiparasitic activity. Compared to the **2a**–**f** thiosemicarbazone analogs, the compounds of series four show a stronger activity, which largely confirms the great importance of the heterocyclic ring. The presence of a substituent, such as nitro or methoxy or bromo groups in the *para* position with respect to the aryl ring in the fifth position of the pyrazole, determined the antiparasitic effect. The conversion of thiosemicarbazone into *S*-methyl and 2-amino-1,3,4-thiadiazole derivatives improved the efficacy, mainly against *Leishmania amazonensis* and also *Trypanosoma cruzi*. It can be safely stated that *S*-methylthiosemicarbazones, such as **3a**, **3d**, **3f** and 2-amino-1,3,4-thiadiazole-pyrazole hybrids (**4a**, **4d**, **4f**) constitute the dominant structures in the context of the synthesis of potential antiparasitic drugs [48].

### 4.3. Derivatives of Aurone

Schistosomiasis is a disease caused by some species of blood trematodes commonly known as blood flukes that are treated by administering praziquantel. Due to the low activity of the substance against juvenile parasites, the variable oral bioavailability of the drug and the risk of resistance, the search for new compounds as an alternative to the current treatment of the disease began. A synthetically derived drug approved by the FDA for the treatment of infections caused by blood flukes is diminazene. Based on research, it was found that this drug has anthelmintic properties and inhibits the risk of liver damage by *Schistosoma* eggs [52].

**Figure 6:**
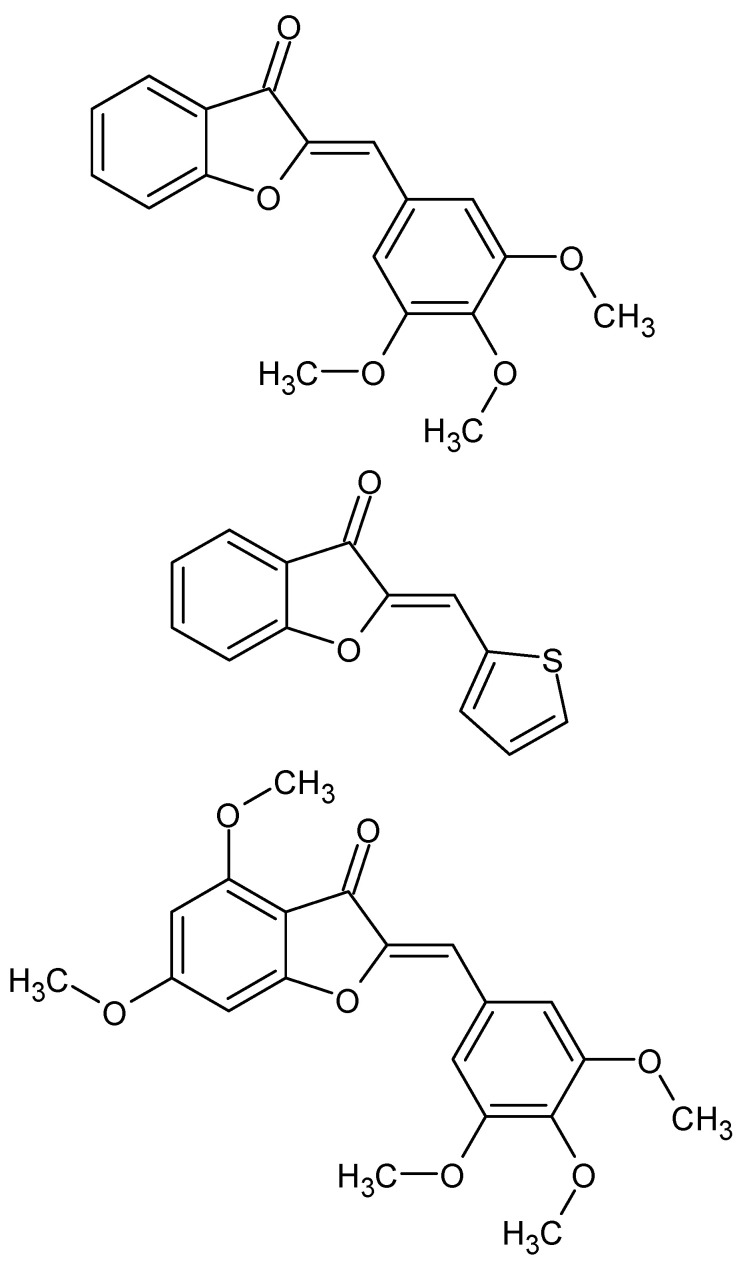
The structures of the three most active aurone analogs.

Pereira et al. proved an interesting antiparasitic effect of new aurone derivatives (Figure 6) against *Schistosoma mansoni*. Aurones are naturally occurring compounds belonging to flavonoids, the antiparasitic activity of which has not been known so far. The compound with the strongest antischistosomal activity is the thiophene derivative of aurone, which, compared to praziquantel, is not cytotoxic to mammalian cells and is effective both in young and adult *Schistosoma mansoni*. The novel aurone analogs, therefore, have great potential for use in *S. mansoni* infections [53].

### 4.4. Analogs of Naphthoquinone

**Figure 7:**
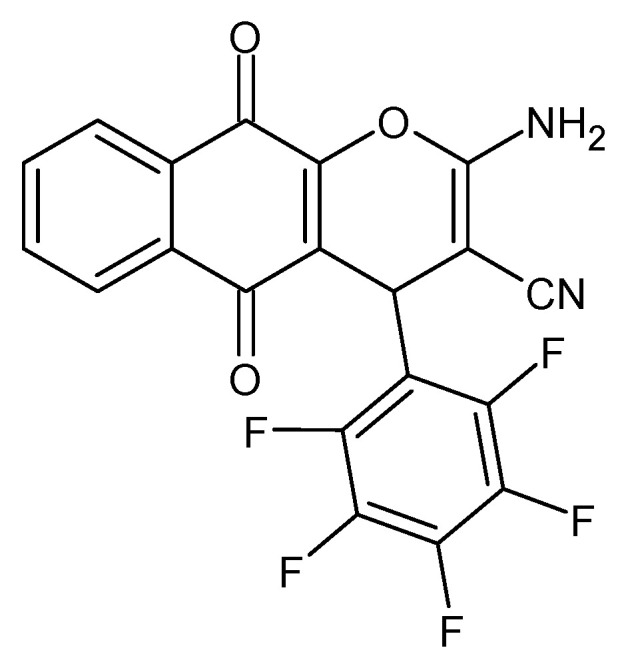
The structure of 2-amino-5,10-dihydro-5,10-dioxo-4-(2,3,4,5,6-pentafluorophenyl)-4H-naphtho[2,3-b]pyran-3-carbonitrile.

New analogs of pyranonaphthoquinone were synthesized to investigate the antiparasitic activity against *Leishmania major*, *Trypanosoma brucei* and *Toxoplasma gondii*. The pentafluorophenyl derivative (Figure 7) showed activity against *Toxoplasma gondii*. In turn, a derivative of 3-chloro-4,5-dimethoxyphenyl, comparable to amphotericin B, turned out to be effective in the control of *Leishmania major* but it was also quite toxic to Vero cells [54].

The growing resistance and toxicity of current drugs are forcing researchers to seek alternative treatment options. According to preclinical studies, substances, such as aminopyrazoles, benzoxaborole and nitroimidazoles show promising antiparasitic activity [55].

**Figure 8:**
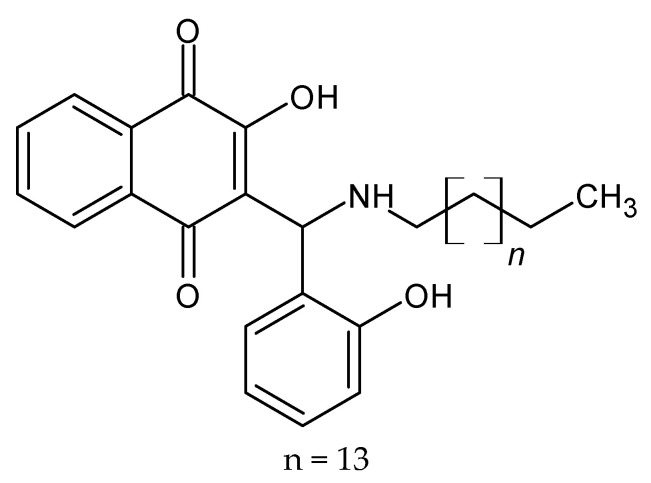
The structure of the new active derivative of naphthoquinone.

A series of new lawsone Mannich bases derived from (halo-)salicylaldehydes and long-chained alkyl amines (C12 and C16 chains) were prepared by Nasr et al. New compounds were tested against the pathogenic parasites *Leishmania major*, *Trypanosoma gondii* and *Typanosoma b. brucei*. One compound: hydrochloride of 3-[(hexadecylamino)(2-hydroxyphenyl)methyl]-2-hydroxy-1,4-naphthoquinone (Figure 8) is a particularly promising new drug candidate for the treatment of toxoplasmosis. The three derivatives 3-[(hexadecylamino)(2-hydroxyphenyl)methyl]-2-hydroxy-1,4-naphthoquinone, 3-[(hexadecylamino)(3,5-dichloro-2-hydroxyphenyl)methyl]-2-hydroxy-1,4-naphthoquinone, and 3-[(hexadecylamino)(5-nitrofuran-2-yl)methyl]-2-hydroxy-1,4-naphthoquinone and its hydrochlorides were also efficacious against *T. b. brucei* [56].

### 4.5. 3-(ω-aminoalkoxy)-1-benzyl-5-nitroindazoles and Furanyl-N-acylhydrazone Derivatives (PFUR)

Metronidazole and tinidazole are popular medications used to treat vaginal trichomoniasis caused by *Trichomonas vaginalis* [57]. Due to the many side effects and increasing parasite resistance, 5-nitroimidazoles can be used as an alternative treatment modality. Among them, the following should be distinguished: paromomycin, furazolidone or nonoxynol-9 [58].

**Figure 9:**
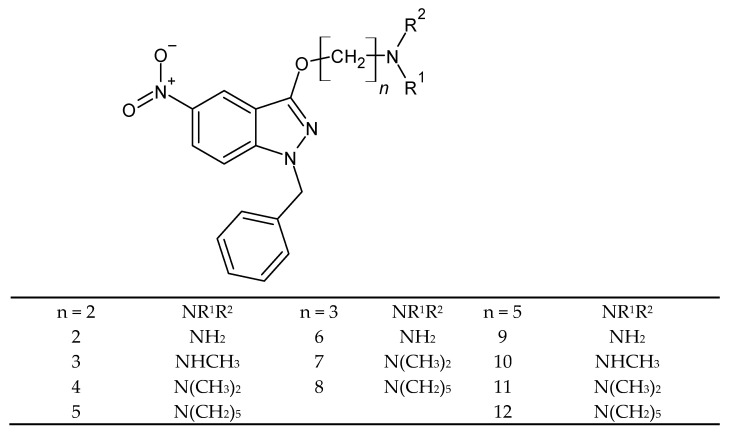
The structure of 3-(ω-aminoalkoxy)-1-benzyl-5-nitroindazoles.

Due to the lower effectiveness of alternative drugs compared to those used as first-line drugs, studies have been carried out to find new molecules in the treatment of *Trichomonas vaginalis* infections. Mirna Samara Dié Alves et al. synthesized and tested 3-(ω-aminoalkoxy) -1-benzyl-5-nitroindazoles (Figure 9) with different substituents on the nitrogen atom of the amino group for antitrichomic activity. The substrate for the syntheses was 1-benzyl-5-nitroindazol-3-ol, which was obtained in the alkylation reaction. The **9**–**11** derivatives with IC_50_ values ≤ 6.5 µM turned out to be the most active compounds. The results of in vitro tests against resistant and susceptible parasites may be the starting point for the synthesis of further indazole analogs [59].

**Figure 10:**
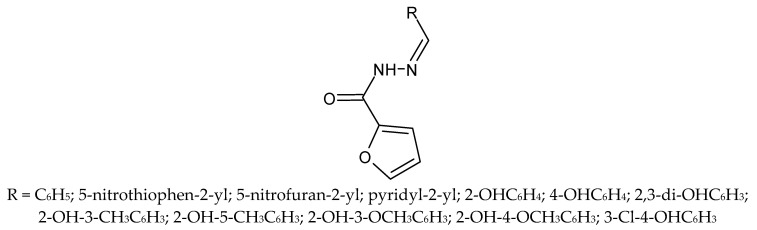
The structure of furanyl-N-acylhydrazone derivatives.

In 2020, Alves et al. published the results of in vitro and in silico activity studies of 12 derivatives of furan-2-yl-N-acylhydrazone (PFUR) against *Trichomonas vaginalis* (Figure 10) [60]. Due to the increasing resistance to metronidazole, there is a need to search for new compounds effective against the above parasite. Furanyl-N-acylhydrazone derivatives have high biological activity: antimalarial, antitumor, antifungal and antibacterial [61,62,63,64]. In addition, furan present in the structure of PFUR compounds has inter alia, antibacterial, antiviral, anti-inflammatory, antifungal, and anticancer properties [65]. The combination of both pharmacophore groups in PFUR compounds grants a chance to obtain new compounds with promising biological activity.

In silico studies have shown that PFUR **4a** and **4b** have high antiparasitic potential. In support of this thesis, new derivatives were synthesized and their activity against *Trichomonas vaginalis* was tested. Compounds **4a** (R = 5-nitrothiophen-2-yl) and **4b** (R = 5-nitrofuran-2-yl) were shown to kill parasites after 24 h of exposure. The IC_50_ values were calculated to be 1.69 µM and 1.98 µM. Both analogs inhibited growth by <20% in CHO-K1 cells, which was comparable to the corresponding drug and a selectivity index > 7.4. The remaining N-acylhydrazone derivatives have shown antiparasitic activity against *T. cruzi* both in vivo and *in vitro*. Among the remaining 10 compounds, PFUR **4a** and **4b** are distinguished by the presence of a nitro group in the heterocyclic ring. Thus, it was found that by introducing a nitro group, it is possible to increase the antiparasitic activity against protozoa [60].

### 4.6. New Analogs of Thiazolidin-4-One

Toxoplasmosis is a disease caused by *Toxoplasma gondii*. In medicine, there are few effective and safe drugs to fight this disease. The increase in drug resistance is also still observed, hence the need to search for new molecules with a more favorable profile of action. The main focus was on thiazolidin-4-one derivatives. Many analogs of this heterocyclic system have a broad spectrum of activity: antidiabetic, antibacterial, antifungal, anticancer and anti-inflammatory [66,67,68,69,70]. Knowledge of the effective antiparasitic activity of thiazolidin-4-one analogs against *T. gondii* has motivated scientists to search for effective derivatives with low toxicity.

**Figure 11:**
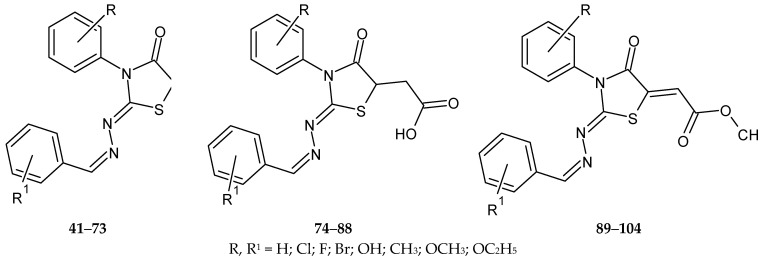
The structure of new thiazolidin-4-one derivatives.

In the first stage, thiosemicarbazones were obtained by condensing thiosemicarbazide derivatives with hydroxybenzaldehydes. In the next stage in the first series of compounds (**41**–**73**) thiazolidin-4-one derivatives were obtained, the second series (**74**–**88**) consisted of (4-oxothiazolidin-5-yl)acetic acid derivatives, and the third series of (4-oxothiazolidine)acid derivatives -5-ylidene)acetic acid (**89**–**104**) (Figure 11) [71].

Compounds **42**–**69** were characterized by high cytotoxicity CC_50_ (50% inhibition of cell proliferation) of 83.54 ± 8.27–385.61 ± 25.60 µM sequentially. The exception was compound **43**, which was 2-{[(4-hydroxyphenyl)methylidene]hydrazinylidene}-3-phenyl-1,3-thiazolidin-4-one. All the above substances were eliminated from further studies. The second group of compounds was characterized by lower cytotoxicity compared to the first group. In this series, CC_50_ values were 427.98 ± 21.90–492.91 ± 19.89 µM. The compounds of series two and three were used to evaluate the inhibition of *Toxoplasma gondii* growth in vitro. Derivatives of (4-oxothiazolidin-5-yl)acetic acid having phenyl substituents in the 3-position of the thiazolidine system turned out to be inactive. The change to a 4-chlorophenyl substituent in position three (**82**–**88**) increased the antiproliferative activity against *Toxoplasma gondii*. The inhibitory concentration (IC_50_) values ranged from 115.92 ± 21.68 to 271.15 ± 24.96 µM. The presence of a chlorine atom in the phenyl ring at position three (**84**–**88**) increased the effects on the parasite (IC_50_ = 129.42 ± 14.14 µM).

Among the last series, derivatives **94** and **95** turned out to be the most active compounds. They showed 392 times better inhibition of proliferation compared to sulfadiazine and 18 times better than the synergistic effect of sulfadiazine and trimethoprim.

For all 12 active compounds (**82**–**88**; **91**–**95**), the selectivity index value was 1.75 to 15.86 (CC_30_/IC_50_). A certain relationship was noted: the methoxy and ethoxy (electron-donating) groups present in compounds **92**, **93** were active at an IC_50_ ~180–190 ± 24.50 µM. However, the lack of a substituent in this fragment, as in the case of compound **91**, increases the activity of the substance, IC_50_ = 92.88 ± 21.91 μM. The introduction of a chlorine or bromine atom, as in the case of compounds **94** and **95**, increases their activity IC_50_~27–28 ± 5.05 μM [71].

### 4.7. Conjugates of Carbohydrates and Naphthalene Diimide

Zuffo et al. published an article on the possibility of developing new antiparasitic drugs against *Trypanosoma brucei*, *Leishmania* spp. In the organisms of these parasites, there are genome encoding sequences that make up the G-quadruplex (G4), which is involved in processes necessary for the survival of the parasite. It was he who became the target of the discovery of new antiparasitic drugs. G-quadruplexes are secondary nucleic acid structures. Guanines form quartets and their connections are strengthened by Hoogsteen’s hydrogen bonds. The main role of the G-quadruplex is the transcription and translation, as well as telomere maintenance [72].

**Figure 12:**
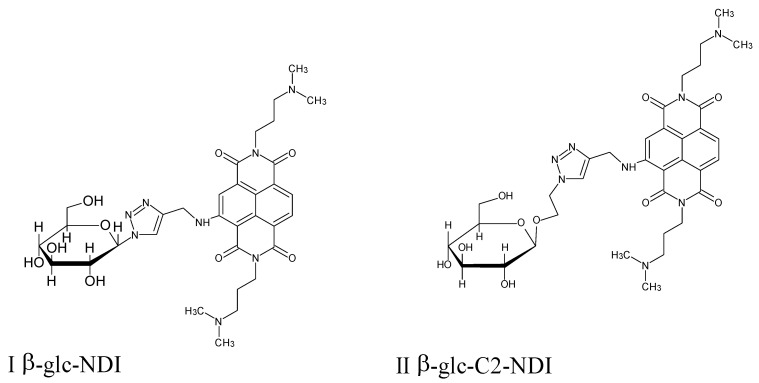

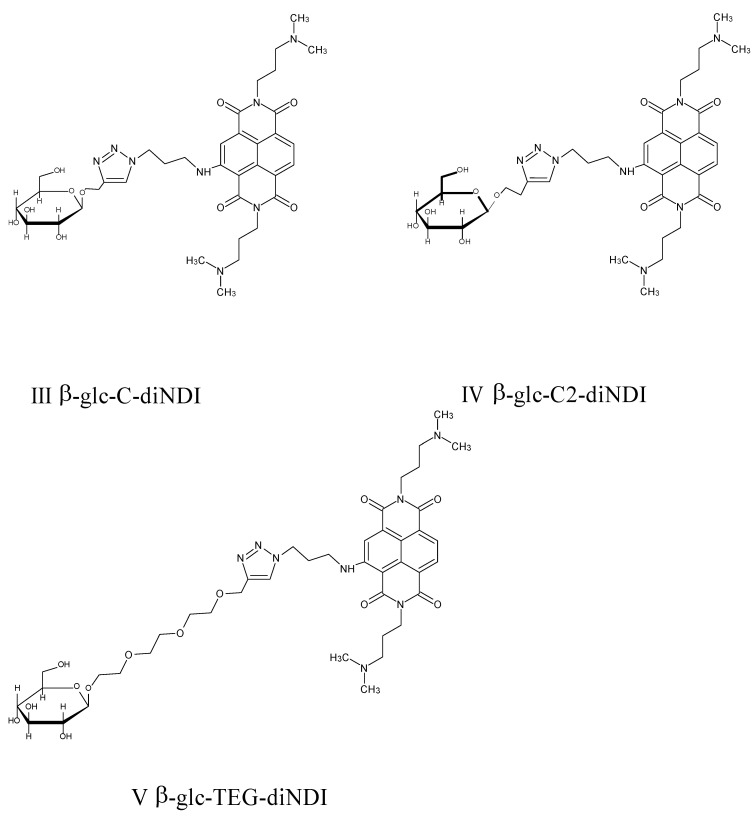
The structure of previously reported conjugates (**I**,**II**) and new conjugates (**III**–**V**).

Earlier studies have synthesized a series of G4 ligands of the carbohydrate-naphthalene diimide conjugate and demonstrated their antiproliferative and antiparasitic activity mainly against *Trypanosoma brucei* [73]. Based on previous research and knowledge gained, a new series of carb-NDIs compounds was obtained. The main structural difference between the previously reported conjugates and the newly synthesized ones is the increased distance between the sugar molecule and the NDI moieties, as well as the inverted triazole system (Figure 12). In addition, the new types of conjugates have a different structure of NDI side chains and the sugar molecules used. An amount of 24 conjugates were obtained and tested for antiparasitic activity against *Trypanosoma brucei* and *Leishmania spp.* For carb-NDI (1–24) conjugates, the IC_50_ value against *T. brucei* ranged from sub-μM 0.04 ± 0.01 to 0.83 ± 0.12 × 10^−6^ M. β-glc-C2-diNDI, β-lac-TEG-diNDI and β-malt-TEG-diNDI turned out to be the most active compounds. On the basis of flow cytometry and confocal microscopy, it was proved that the conjugates are very well absorbed by parasites and are mainly located in the nuclei and kinetoplasts. Thanks to sugar groups, these compounds are perfectly absorbed by parasites. Properly obtained selectivity indices and the lack of hemolytic activity offer great hope and possibilities for the synthesis of carb-NDIs conjugates against *Trypanosoma brucei* [72].

### 4.8. Omega-3 Fatty Acids

New scientific reports revealed the inhibitory effect of omega-3 fatty acids on the proliferation and survival of *Toxoplasma gondii*. They work through AMPK-dependent autophagy. It has been found that omega-3 fatty acids may be an interesting route for the synthesis of compounds that prevent toxoplasmosis. At this stage of considerations, it is necessary to conduct further studies for the clinical evaluation of omega-3 fatty acids and to identify the most effective combinations with other drugs in the treatment of toxoplasmosis [74].

### 4.9. Miltefosine and Other Metacaspase Activators and Inhibitors

Metacaspases are cysteine proteases that have the catalytic Cys-His dyad and are found in fungi, protozoa and plants. Metacaspases are potential candidates as antiparasitic drugs. These unique proteases induce apoptotic cell death and regulate the stress factors of various protozoa. The specificity of the substrate catalytic site and the lack of physiological occurrence in humans make them a promising target for new drugs [75].

Metacaspases have been divided into three types, differing in their structure, and with the evolution, additionally, the subtypes of their main division. Type I metacaspases are characterized by the presence of an N-terminal extension and the presence of a zinc finger. Type II has a linker separating the p20 and p10 domains. Type III metacaspases are distinguished by the location of the p10 domain N-terminal to the p20 catalytic domain [76].

During studies of metacaspases of specific parasites, it was noticed that *Trypanosomal* and *Plasmodium* metacaspases cause programmed cell death, cell proliferation and parasite cytokinesis [77]. *Leishmania* metacapases are responsible for the regulation of stress and parasite autophagy [78].

**Figure 13:**
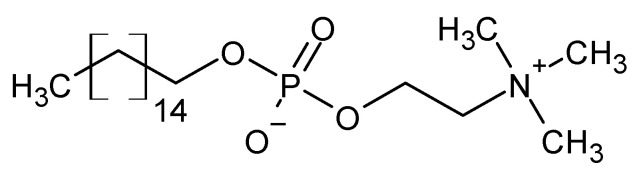
The structural formula of miltefosine.

**Figure 14:**
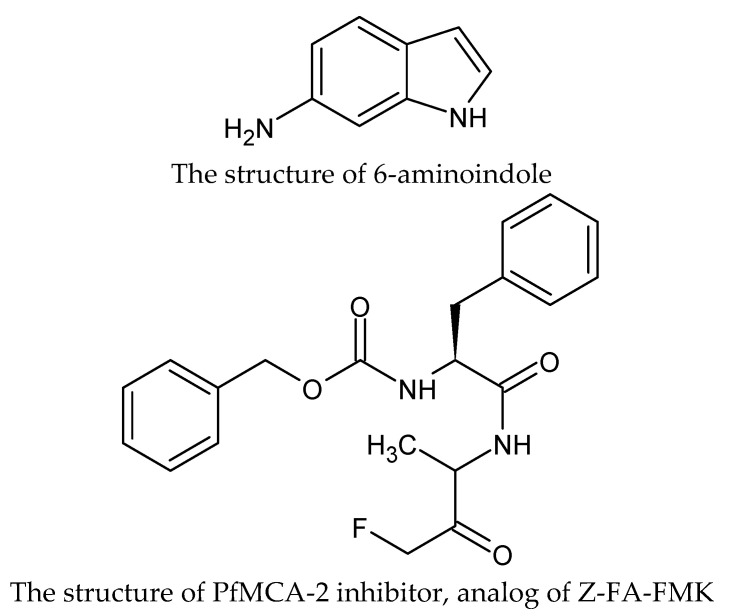
The structure of potential inhibitors of protozoan metacaspases.

The currently used drug in the treatment of leishmaniasis is miltefosine (Figure 13), the action of which, due to the activation of metacaspases, induces programmed cell death [79]. It has also been proved that specific metacaspase inhibitors (Figure 14) reduce the lifespan of the parasite. As a result, both activators and specific metacaspase inhibitors represent a potential target for new antiparasitic drugs [80].

### 4.10. 1,3,4-Thiadiazole Derivatives

**Figure 15:**
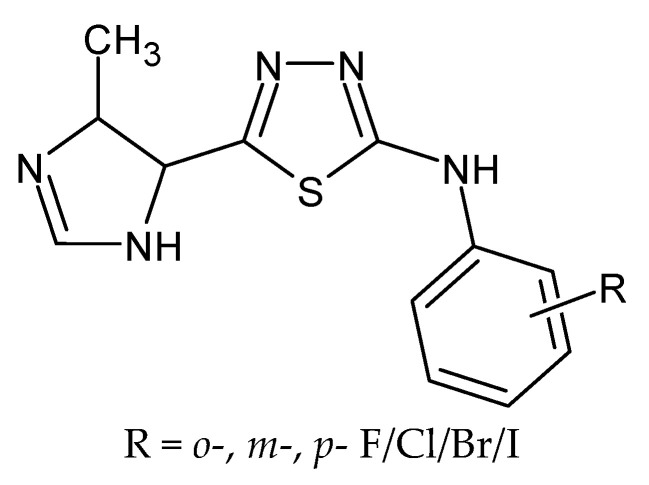
The structure of 1,3,4-thiadiazole derivatives.

Weglinska et al. obtained a series of novel 1,3,4-thiadiazole-2-halophenylamines (Figure 15) functionalized at the C5 position with the 4-methylimidazole ring and screened for their effects on *Toxoplasma gondii* host cell invasion and proliferation. Results showed that all of them compare favorably to the control drugs sulfadiazine and trimethoprim in terms of *T. gondii* growth inhibition and selectivity toward the parasite. The most potent five compounds (with *meta*-fluoro, *meta*-chloro, *meta*-bromo, *meta*-iodo and *para*-iodo substitution) were tested for their efficacy in inhibition of tachyzoites invasion and subsequent proliferation. All derivatives significantly inhibited the parasite invasion and intracellular proliferation via direct action on both tachyzoites and parasitophorous vacuoles formation. The most effective was a derivative with the *para*-iodo substituent that caused a reduction in the percentage of infected host cells by 44% and the number of tachyzoites per vacuole by 93% compared with non-treated host cells. Presented results indicate that 1,3,4-thiadiazoles could be considered as lead compounds for the future design of anti-*Toxoplasma* agents [81].

### 4.11. Thiazole Derivatives

**Figure 16:**
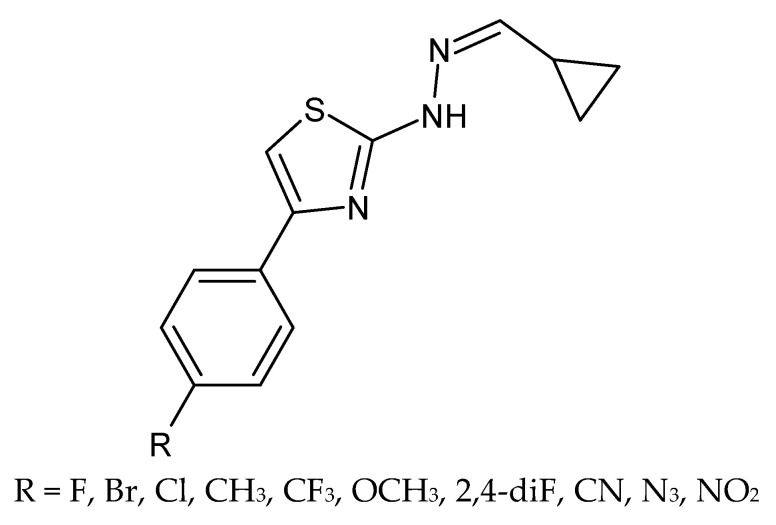
The structure of thiazole derivatives.

Łączkowski et al. presented synthesis and investigation of antifungal, anticonvulsant and anti-*Toxoplasma gondii* activities of ten novel (2-cyclopropylmethylidene)hydrazinyl)-thiazole derivatives (Figure 16). Some of them, with fluoro, cyano and nitro group in position *para* of the phenyl ring, showed significant anti-*Toxoplasma gondii* activity, with IC_50_ values 31–52 times lower than those observed for sulfadiazine. The results of the cytotoxicity evaluation and anti-*Toxoplasma gondii* activity studies showed that *Toxoplasma gondii* growth was inhibited at non-cytotoxic concentrations for the mouse L929 fibroblast and the African green monkey kidney (Vero) cells [82].

**Figure 17:**
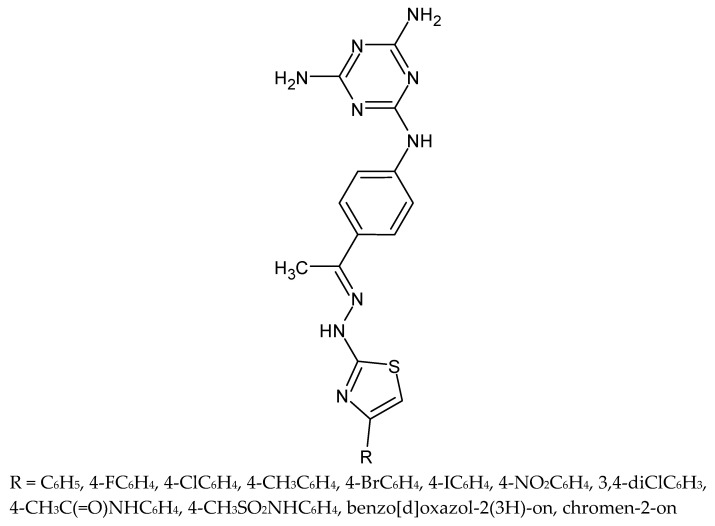
The structure of 2,4-diaminotriazine-thiazole derivatives.

The same authors presented a synthesis, characterization and investigation of antiproliferative activities against human cancer cell lines and *Toxoplasma gondii* parasite of twelve novel 2,4-diaminotriazine-thiazoles (Figure 17). Similarly, as in the case of the previous compounds, the new derivatives were characterized by a much lower activity (IC_50_ values 9–68 times lower) than sulfadiazine used as a reference drug [83].

### 4.12. Thiosemicarbazide Derivatives

Thiosemicarbazides are one of the most promising groups of compounds showing significant antiparasitic activity.

**Figure 18:**
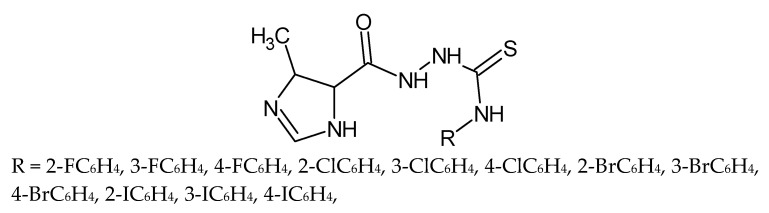
The structure of 1-(4-methylimidazol-5-oyl)-4-substituted thiosemicarbazide.

Paneth et al. described the synthesis and anti-*Toxoplasma* activity of new 4-arylthiosemicarbazides with a five-membered heterocyclic ring—4-methylimidazole at the N1 position (Figure 18). The best active compounds were two derivatives with 3-nitrophenyl and 4-nitrophenyl substituents in position four of the thiosemicarbazide skeleton. These substances were 125 to 184 fold more potent than the reference drug sulfadiazine, respectively [84,85].

**Figure 19:**
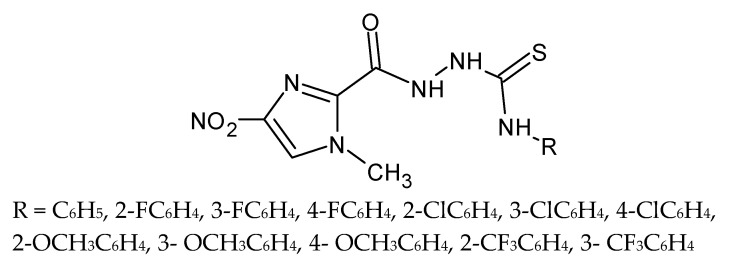
The structure of 1-(1-methyl-4-nitroimidazol-2-oyl)-4-substituted thiosemicarbazide.

The 1-methyl-4-nitroimidazole-2-carbohydrazide was a starting material for the synthesis of new thiosemicarbazide derivatives (Figure 19). All compounds were screened for their antiparasitic activity. Most of the compounds tested showed very good nematicidal activity. Selected compounds may become potential candidates for anthelmintic drugs. The most potent of them with phenyl, *ortho*-chlorophenyl and *meta*-chlorophenyl substituents were more active than albendazole [86].

**Figure 20:**
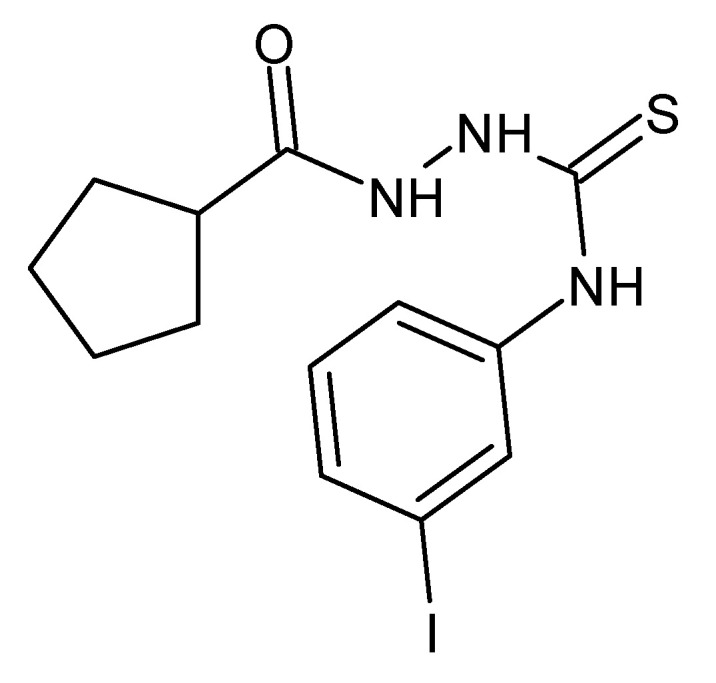
The structure of 1-cyclopentanoyl-4-(3-iodophenyl)thiosemicarbazide.

In 2021, Bekier et al. reported anti-proliferation effects of 4-arylthiosemicarbazides, with a cyclopentane substitution at the *N*1 position on the highly virulent RH strain of *Toxoplasma gondii*. The highest in vitro anti-*Toxoplasma* activity was found in the *meta*-iodophenyl derivative (Figure 20). In silico experiments demonstrated inhibitory effects of thiosemicarbazides on tyrosinase (Tyr) activity, and a good correlation was found between the percentage of Tyr inhibition and IC_50*Tg*_. This may be a new direction in the search for effective drugs against toxoplasmosis [87].

## 5. Conclusions

Basic approaches to discovering a drug for tropical diseases can be classed as short-to-medium term (based on known compounds or compound classes) or long-term (requiring discovery of new chemical substances).

The first strategy includes the use of several known drugs simultaneously. The combinations of existing drugs offer possibilities of synergy, reduced toxicity and slowing the development of resistance. Furthermore, new indications for existing drugs are an attractive strategy that offers great savings in time and money. For example, miltefosine, which originally was used as an anticancer drug in animals (1987), was approved in India in 2002 as an anti-leishmaniasis drug.

In the medium term, improvement of known drugs and classes of compounds by synthesizing their analogs may prove to be effective. The changes are aimed at improving activity, pharmacokinetic properties and reducing toxicity.

Another approach to finding new drugs is screening compound libraries. An alternative and arguably a more productive one is to screen focused sample collections. Here, the emphasis is on identifying compounds with defined biological effects against related parasites, activity against isoenzymes or receptors related to known molecular targets of other organisms.

A final approach is de novo drug discovery. Strategies aim to discover novel active compounds unrelated to the known drugs. Modern medical chemistry is dominated by the drug design approach based on a known molecular target, but for parasitic diseases, de novo synthesis and the analysis of compounds in terms of their action on whole parasites is still valid.

Currently, 272 clinical trials on parasitic diseases are being carried out. Only five of them concern the use of new substances (Table 3).

In this short review, we presented the current strategies to search for new antiparasitic drugs.

When analyzing new structures, compounds with very different structures show promising antiparasitic activity, but it seems that the most promising are thiosemicarbazide derivatives, characterized by high activity in both *Toxoplasma gondii* and nematodes of the genus *Rhabditis* sp. This is an important course of action. Most clinical trials are focused on malaria, without other parasitic diseases, which are also widespread in highly developed countries.

## Figures and Tables

**Table 1:** The structure of six new linezolid analogs.

Linezolid	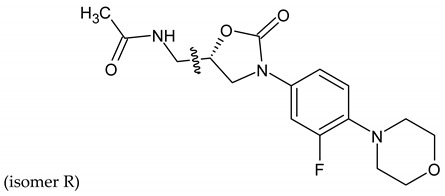 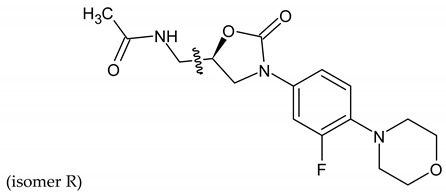		
**4** isomer S	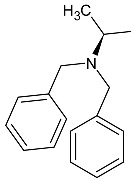	**5** isomer R	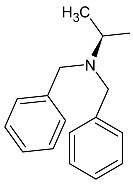
**6** isomer S	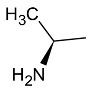	**7** isomer R	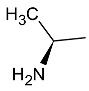
**8** isomer S	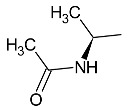	**9** isomer R	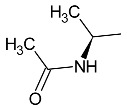

**Table 2:** The structures of new analogs of trifluoromethylated hybrids of pyrazole.

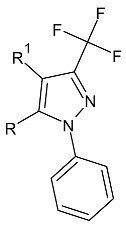		
	R	R^1^
**2a**–**f**	4-nitrophenyl (**a**); 4-fluorophenyl (**b**); 4-chlorophenyl (**c**); 4-bromophenyl (**d**); phenyl (**e**); 4-methoxyphenyl (**f**)	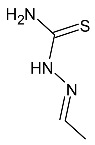
**3a**–**f**	4-nitrophenyl (**a**); 4-fluorophenyl (**b**); 4-chlorophenyl (**c**); 4-bromophenyl (**d**); phenyl (**e**); 4-methoxyphenyl (**f**)	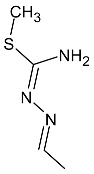
**4a**–**f**	4-nitrophenyl (**a**); 4-fluorophenyl (**b**); 4-chlorophenyl (**c**); 4-bromophenyl (**d**); phenyl (**e**); 4-methoxyphenyl (**f**)	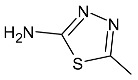

**Table 3:** The structure of new antiparasitic compounds in clinical trials.

Symbol of Compound	Chemical Structure	Disease
Zy-19480	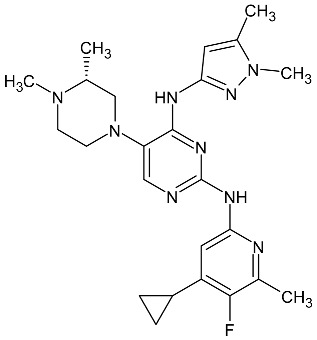	Malaria
SJ733	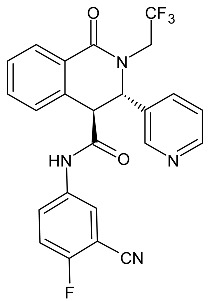	Malaria
KAE609	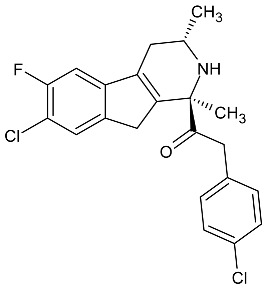	Malaria
AWZ1066S	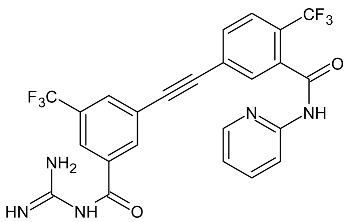	Filarial diseases
ABBV-4083	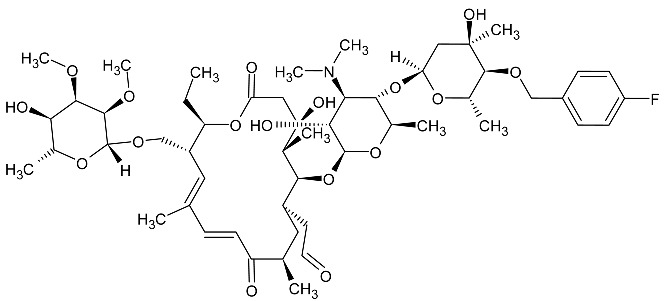	Filarial diseases

## Data Availability

Not applicable.
